# Frequency of Vitamin-D deficiency in children with Urinary tract infection: A descriptive cross-sectional study

**DOI:** 10.12669/pjms.37.4.3896

**Published:** 2021

**Authors:** Saba Qadir, Shazia Memon, Muhammad Nadeem Chohan, Yasmeen Memon

**Affiliations:** 1 Dr. Saba Qadir, MBBS, FCPS. Medical Officer, Department of Pediatrics, Liaquat University of Medical and Health Sciences, Hyderabad, Sindh, Pakistan; 2 Prof. Shazia Memon, MBBS, FCPS. Department of Pediatrics, Liaquat University of Medical and Health Sciences, Hyderabad, Sindh, Pakistan; 3 Dr. M. Nadeem Chohan, MBBS, FCPS. Assistant Professor, Department of Pediatrics, Liaquat University of Medical and Health Sciences, Hyderabad, Sindh, Pakistan; 4 Dr. Yasmeen Memon, MBBS, FCPS. Professor of Pediatrics, Isra Medical University, Hyderabad, Sindh, Pakistan

**Keywords:** Children, Urinary Tract Infection, Vitamin D deficiency

## Abstract

**Objective:**

To determine Vitamin-D status in children with urinary tract infection.

**Methods:**

A Cross-sectional study was done at Pediatric Department, Liaquat University Hospital Hyderabad, from July 2019 to March 2020. A total of 172 children of either gender from 2 to 60 months of age with confirmed urinary tract infection (UTI) (having positive urine C/S report) were included in the study. The child who received antibiotics 48 hours prior or already on immunosuppressive drugs and steroids from previous health record or by taking clinically relevant history), children with CKD on vitamin-D supplementation, and known case of Vitamin-D deficiency were also excluded from the study. All study participants were evaluated for vitamin-D level by high performance liquid chromatography. Urine sample was collected for C/S and 1 cc venous blood was taken for Vitamin D status (ng/ml). The mean ± standard deviation (SD) and stratification was calculated for age, duration of urinary tract infection and vitamin-D level. Post stratification chi-square test was applied for all categorical variables at 95% confidence interval (CI) and P-value ≤0.05 was considered significant.

**Results:**

The average age of the patients was 41.51±18.34 months. There were 130 (75.58%) females and 40 (23.25%) males. Most common complaint of the children was fever 150 (87.21%). Vomiting was present in 31 (18.02%), abdominal pain 22 (12.79%) and dysuria in 15 (8.72%) children. A total of 129 (75%) children had pyelonephritis and 15 (25%) had cystitis. (Frequency of vitamin-D deficiency in children with diagnosed UTI was 45.93% (79/172). Mild vitamin D deficiency was present in 42 (53.16%) children, while moderate deficiency in 55 (69.62%) children. *E. Coli* was the most common pathogen in both mild and moderate vitamin D deficiency i.e., 20 (47.61) and 31 (56.36%) respectively.

**Conclusion:**

The frequency of urinary tract infection is more common in children having vitamin D deficiency.

## INTRODUCTION

Vitamin-D has a vital role in the immune regulation, and it has a systemic effect on pathogens.^1^,^2^ Vitamin D deficiency can cause hypocalcemia that reduces the function of neutrophils and lymphocytes.^3^ There are various functions of Vitamin-D including suppression of cytokines, like suppuration of cytokine (IL6, IL 8) and inflammatory cascade taking place after attachment of pathogen to uroepithilium, thereby it may limit the severity of UTI 4 hence suppress the effect of infection. Deficiency of vitamin-D is associated with sepsis, pneumonia, and influenza.^5^ In previous studies Vitamin-D deficiency was associated with UTI; 6 having the prevalence of 20%.^7^

UTI is a common diagnosis in children. It can cause abdominal pain, fever, frequency, dysuria and hematuria. In the first year of life UTI is most common in boys than girls but afterwards it is more common in girls. There are various risk factors for UTI like congenital anomalies of kidneys and urinary tract (PUV and VUR), constipation and bladder dysfunction. Prevalence of UTI in girls is 3-5% and 1% in boys.^8^ About 5% of febrile girls in infancy presents with UTI, while 20% of uncircumscribed boys present with UTI. Escherichia coli infection is the most common pathogen (80% to 90%) causing the UTI.^9^

Vitamin-D Supplementation before and during the episode of urinary tract infection can improve the clinical status.^10^ Vitamin-D Supplements are available in market at reasonable price. UTI can be managed more appropriately by using both vitamin-D supplements and antibiotics. This practice may decrease the misuse of antibiotics hence reducing the cost of management. Epithelium derived cathelicidin is the first line defense mechanism against the bacteria’s causing UTI. Cathelicidin is produced by epithelium of urinary tract during infection.^11^ Cathelicidin has a defined vitamin-D dependent mechanism. Bacterial adherence to urinary tract mucosa leads to the production of cathelicidin.^12^ Normal Vitamin-D levels in serum is mandatory for the optimal cathelicidin production by macrophages.^13^

There is no local study that has assessed the impact of vitamin-D level on UTI, thus this study is relevant and specific to generate local data by evaluating the magnitude of vitamin-D deficiency in children with UTI and to compare our results with international literature. Our objective was to find out the frequency of vitamin-D deficiency in children with diagnosed UTI.

## METHODS

This cross-sectional study was conducted at Pediatric Department, Liaquat University Hospital, Jamshoro/Hyderabad by from 02-07-2019 to 14-03-2020. Non probability consecutive sampling technique was used and sample size was calculated by taking Vitamin-D deficiency prevalence in urinary tract infection as 20%;^7^ with 6% margin of error. Total 172 children with urinary tract infection were taken in this study.

All children of two to sixty months of age, of either gender with suspicion of urinary tract infection based on clinical features (like dysuria, hematuria, abdominal pain, pack pain, increased frequency of urine, history of fever), were worked up by urinalysis and urine culture and sensitivity. Urine CS positive (>10^5^ colonies of a single urinary tract pathogen / ml of urine specimen) were included in the study. Children with malnutrition and other systemic illness were excluded. The child who received antibiotics 48 hours prior or already were on immunosuppressive drugs and steroids from previous health record or by taking clinically relevant history), children with CKD on Vitamin-D supplementation, and known case of vitamin-D deficiency were also excluded from the study.

Children presenting to pediatric out door department with suspicion of urinary tract infection were recruited in study by taking consent from attendant / guardian of the children and by collecting urine sample of around 10 ml under aseptic measures in sterile urinary bottle and send to laboratory for analysis. Urine specimen was taken by urinary catheterization in all children If urine sample was not analyzed within 10 minutes, then it was refrigenetad and analyzed later on. All children with suspected urinary tract infection whose urine C/S report became positive were evaluated and explored in laboratory for Vitamin-D deficiency (≤ 20 ng/ml) (by taking one cc venous blood in a sterilize syringe. Vitamin-D was measured by high performance liquid chromatography. All the specimens were examined by senior pathologist of diagnostic and research laboratory, having more than three years’ clinical laboratory experience. Data collection like Urine C/S and Serum vitamin D level and all such maneuvers (relevant history, specific clinical examination and sampling) was performed by principal researcher. All expenses were born by the researcher. This study was done after the approval of CPSP (CPSP/REU/PED-164-3854).

Serum vitamin-D level ≤ 20 ng/ml was labelled as vitamin-D deficiency.^14^ Urinary tract infection was labelled when urine culture showed growth of >10^5^ colonies of a single urinary tract pathogen / ml of urine specimen

SPSS version 22.00 was used for data analysis. The frequency and percentage (%) were calculated for age, gender, fever, and vitamin-D deficiency. The mean ± standard deviation (SD) and stratification was calculated for age, duration of urinary tract infection and vitamin-D level.

Post stratification chi-square test was applied for all categorical variables at 95% confidence interval (CI) and P-value ≤0.05 was considered significant.

## RESULTS

Total 172 children with confirmed UTI were included in this study. Baseline characteristics are shown in Table-1. The average age of the patients was 41.51±18.34 months and mean vitamin-D level was -20.33 SD ±15.0---. There were 132(76.74%) male and 40 (23.6%) females. Most of the children had fever 150 (87.21%) (Table-I). Frequency of vitamin-D deficiency in children with diagnosed UTI was 45.93% (79/172). It was not statistically significant among age groups (p=0.637). It was significantly high in female as compare to male (60% vs. 41.7%; p=0.042) It was not statistically significant with fever as shown in Table-II. Mild vitamin D deficiency was present in 42 (53.16%) children, while moderate deficiency in 55 (69.62%) children. E Coli was the most common pathogen in both mild and moderate vitamin D deficiency ie 20 (47.61) and 31 (56.36%) respectively (Table-III).

**Table-I:** General characteristics of study participants (n=172).

Characteristic	N	%

	Mean	Standard Deviation
Age	41.51	18.34
<12 (months)	34	19.77
13-48 (months)	73	42.44
>48 (months)	65	37.79
***Gender***		
Male	132	76.74
Female	40	23.26
***Clinical Features***		
Fever	150	87.21
Vomiting	31	18.02
Abdominal pain	22	12.79
Dysuria	15	8.72
***Vitamin D Status***		
Mean	20.33	SD ±15.0
Mild Deficiency	42	53.16 %
Moderate Deficiency	55	69.62%

**Table-II:** Vitamin-D status in children with UTI (n= 172).

Age Groups (months)	Vitamin-D status		Total	P-Value

	VD deficient Yes N=79(45.93%)	Normal VD No N=93		
≤ 12	18(52.9%)	16(47.1%)	34	
13-48	33(45.2%)	40(54.8%)	73	0.637
>48	28(43.1%)	37(56.9%)	65	
Gender				
Male	55(41.7%)	77(58.3%)	132	
Female	24(60%)	16(40%)	40	0.042
Fever				
Yes	69(46%)	81(54%)	150	0.962
No	10(45.5%)	12(54.5%)	22	
Duration of UTI				
≤5 days	52(47.7%)	57(52.3%)	109	0.539
> 5 days	27(42.9%)	36(57.1%)	63	0.531

**Table-III:** Pathogens according to Vitamin D Status.

Pathogens	Number	%
Mild Deficiency	42	
Escherichia. Coli	20	47.61
Klebsiella	10	23.80
Enterococcus	04	9.52
Staphylococcus. Aureus	02	4.76
Pseudomonas	06	14.28
Moderate Deficiency	55	
Escherichia. Coli	31	56.36
Klebsiella	08	14.54
Enterococcus	07	12.72
Staphylococcus. Aureus	01	1.81
Pseudomonas	08	14.54

## DISCUSSION

Vitamin-D provides immunity against foreign invaders due to widespread presence of vitamin-D receptors. It has an important role in infectious diseases. In this present study vitamin-D level was positively correlated with UTI in children. Treatment of UTI is challenging due to antibiotics resistance, side effects of antibiotics, and recurrence of infection.

In this study the average age of the patients was 41.51±18.34 months. There were 76.74% male and 23.6% female. Most of the children had fever. In Merrikhi et al. study,^15^ there were more girls 31 (93.9%) presented with urinary tract infection, with a mean age of 5.97 ± 2.90 years.

A recent study reported that in vitamin-D deficient children, 76 (63%) had UTI and 44 had no UTI.^16^ In our study frequency of vitamin-D deficiency in children with diagnosed UTI was 45.93%. In another study children with UTI 12 (33.3% had vitamin-D levels between 12-19 ng/mL^17^ El-Mazary et al. found no differences in the frequency of UTI in infants who receive Vitamin-D supplement and those who have not received supplement.^18^ Nielsen et al found that children who had recurrent UTI also had vitamin-D deficient.^19^

Various studies done to know whether vitamin-D supplement can decrease the risk of urinary tract infection or not, the results are conflicting. In a study, Vitamin-D supplement increased the risk of UTI, with a relative risk of 1.76 (1.07-2.91, *P* < 0.05) 20 Author could not search how the vitamin D supplement increases the chances of UTI. Jianhuan Yang et al. concluded in their study that upper urinary tract infection had lower vitamin-D level than those having lower UTI. Serum vitamin-D <20ng/mL was positively associated with the risk of UTI. Vitamin-D supplementation was associated with a decreased risk of UTI.^21^

In this study fever was the most common complaint 50 (87.21%) in children with UTI having vitamin-D deficiency. The age between 13-48 months was the most common age 73 (42.44%) who presents with UTI. In a meta-analysis Vitamin-D deficiency was associated with increased risk of UTI.^22^ In another study vitamin-D level was lower in the children with UTI.^23^

### Limitations of the study

This was a single center study; multicenter study should be conducted to make results more robust.

## CONCLUSIONS

The results of this study conclude higher frequency of vitamin-D deficiency in children having UTI. Children having urinary tract infection should be assessed for vitamin-D level as deficiency could be corrected and recurrent UTI can be prevented.

### Suggestions

Further studies are needed to assess whether correction of vitamin D may prevent UTIs in children. We included children having the first episode of UTI, children having recurrent UTI should also be assessed.

### Authors’ Contributions:

**SQ, MNC, YM** conceived, designed and did statistical analysis & editing of manuscript.

**SQ, SM, MNC** did data collection and manuscript writing.

**SQ, SM, MNC, YM** did review and final approval of manuscript.

**MNC** responsible and accountable for the accuracy or integrity of the work.
